# Impact of Mn/Co substitution on magnetoelectric and structural properties of ZnO nanostructures thin films

**DOI:** 10.1016/j.heliyon.2025.e42337

**Published:** 2025-01-28

**Authors:** Ángela P. Lanchero, Lina F. Prieto, Heiddy P. Quiroz, Jorge A. Calderón, A. Dussan, F. Mesa

**Affiliations:** a Universidad Nacional de Colombia - Bogotá, Dpto. de Física, Grupo de Materiales Nanoestructurados y sus Aplicaciones, Cra. 30 No. 45-03, Edificio 404 Yu Takeuchi Lab, 121C/121B-1, Ciudad Universitaria–Bogotá, 110001, Colombia; b Grupo de investigación en Bioingeniería, Nanotecnología y Trasferencia de Tecnología, Cluster in Convergent Sciences and Technologies, Universidad Central, Colombia; c Facultad de Ingeniería y Ciencias Básicas, Fundación Universitaria Los Libertadores, Cra.16 # 63a-68, Bogotá, Colombia

**Keywords:** Spintronics, Magnetoelectric effects, Semiconductor device, Thin films

## Abstract

This work investigates the impact of Mn and Co doping on the structural, morphological, electrical, and magnetic properties of ZnO thin films deposited via DC magnetron co-sputtering. Doping concentration, substrate temperature, and substrate type (soda-lime glass and oriented silicon wafer) were systematically varied for potential spintronic applications. X-ray diffraction (XRD) and Raman spectroscopy confirmed the formation of a hexagonal wurtzite crystalline structure with a preferential [002] growth orientation when Mn was incorporated into the ZnO matrix. Raman analysis also ruled out the presence of secondary Co oxide phases in ZnO:Co samples. Films doped with Mn at 25 W exhibited compressive stress of −0.345 %, which increased to −2.03 % at 50 W, highlighting the dopant's impact on lattice strain. FTIR spectra revealed characteristic bands of ZnO:Co, indicating successful incorporation of Co ions into the matrix. SEM and magnetic force microscopy (MFM) showed granular surface morphology and cluster formation at higher Mn concentrations (50 W). Electrical measurements revealed unipolar and bipolar resistive switching (RS) behaviors, associated with the Schottky barrier model, and strongly influenced by substrate temperature and doping levels. Notably, samples doped with Co at 50 W exhibited enhanced interfacial RS properties. Vibrating sample magnetometry (VSM) demonstrated room-temperature ferromagnetic hysteresis in films synthesized at T*s* = 423 K, with Mn (25 W) and Co (50 W) doping. These findings validate the potential of ZnO:Mn/Co as a dilute magnetic semiconductor (DMS) for spintronic applications, offering tailored magnetic and resistive properties through precise control of doping and synthesis parameters.

## 1 Introduction

Nanostructured materials constitute a current field of research aimed at bolstering technological advancements, significantly impacting industrial processes and societal evolution. The study of the physicochemical properties of nanoscale materials has made it possible to find metals, alloys and semiconductors with application opportunities in the development of modern tools and devices such as quantum computers [1] and memory storage of information that advantage the properties of the electron for their operation.

Particularly, ZnO thin films doped with transition elements have garnered significant attention due to their physical and chemical properties for several applications: sensors [1], solar cells [2], magnetism [[2], [3], [4]], structural order [5], optoelectronics [6], spintronics [3,4,[7], [8], [9], [10]], among others [11,12]. The variation of the physical properties of the ZnO material is determined by manufacturing factors such as the synthesis techniques, the substrate temperature and post-annealing processes, substrate type, morphology, and dopant concentration, such as, Al, Ni, Y, Mn or Co [4,11,[13], [14], [15]].

Recently, it has been shown that doping can change the optical, morphological, and structural properties of ZnO. With the incorporation of Ni and Sr it has been shown that there is a strong modification of the diamagnetic behavior on the structure of ZnO [16,17], in turn, when it is doped with Fe^3+^ and Y^3+^ in the central sites of Zn, it alters the deformation reticular on ZnO [18]. It was also found that the average crystallite size, lattice strain, and band gap are influenced by the Er^3+^ dopant ions (3.24–3.27 eV), indicating that the material has exceptional photocatalytic activity to eliminate dyes and drugs from the solution [19]. A study by Zheng-bin Gu et al. [20] indicates that thin films of co-doped ZnO (Mn, Co) on sapphire (0001) substrates exhibit a relatively low gap of around 3.0 eV, compared to ZnO without any impurity atoms which can demonstrate a value of 3.22 eV [21]. Additionally, they showed an increase in the gap when introducing Mn^2+^ ions into the ZnO matrix, while when doped with *Co*^2+^ ions, this gap decreases, showing the effect of the ions on ZnO. Additionally, in this study, the red shift of the band gap indicates enhanced spin exchange interaction in the ZnO:Mn and ZnO:Co films. In addition to the ZnO host phonons, there are four additional vibrational modes at 276.6, 525.6, 634.6, and 643.9 cm^−1^ in the Raman spectra of the thin films, however, the origin of the 634.6 cm^−1^ vibration mode is not identified [20].

In various studies, the coprecipitation method produces pure ZnO nanoparticles and ZnO co-doped with Co/Mn [[22], [23], [24]]. This method, considered within the chemical synthesis methods, allows the dissolution of a soluble element during the formation of a precipitate, obtaining a hexagonal wurtzite structure in a state of the material without impurities. Likewise, in the optical properties of the samples, it is reported from the spectrophotometry measurements that the absorption spectra showed a decrease in the optical energy band as the Mn concentration decreased.

In this work, thin films of ZnO:Mn/Co were synthesized using the DC-magnetron co-sputtering method. This versatile technique is widely recognized for its ability to deposit a broad range of materials, offering high reproducibility and precise control over deposition rates and concentrations. Moreover, it enables the use of temperature-sensitive substrates and stands out as a reliable method for fabricating high-quality, pure thin films.

The choice of dopant concentrations for the fabrication of ZnO:Mn and ZnO:Co thin films was guided by the need to ensure the formation of a dilute magnetic semiconductor (DMS) while avoiding the emergence of secondary phases. Achieving this balance is critical for maintaining the wurtzite crystalline structure of ZnO and integrating the magnetic functionality introduced by the transition metal dopants. Raman spectroscopy measurements confirmed the presence of ZnO as the primary phase, without evidence of secondary Mn or Co oxides. Additionally, the spectra revealed lattice defects, which can be attributed to the substitutional incorporation of Mn or Co into the ZnO matrix. These observations validate the selected doping levels, which ensure the structural integrity of the ZnO matrix and enhance its magnetic and electronic properties, aligning with the material's potential for spintronic applications.

Magnetic measurements indicated a soft ferromagnetic trend in samples doped with low concentrations of Mn, exhibiting a critical temperature of approximately 300 K. In contrast, this effect was not observed in Co-doped samples. This ferromagnetic behavior at or above room temperature and its resistive switching characteristics present promising prospects in spintronic applications.

## 2 Experimental

Thin films of Co/Mn doped ZnO were synthesized using DC magnetron sputtering on various substrate types: soda-lime glass and Silicon wafer (Si n-type). All Glass substrates were cleaned following a standardized process: Soap bath, Alconox detergent, 15 min of ultrasonic bath, distilled water washed, acetic acid (CH_3_COOH) bath, hydrogen peroxide (H_2_O_2_) bath, 15 min of ultrasonic bath in deionized water, isopropanol washed and N_2_ drying. Samples were deposited in argon atmosphere at a work pressure of 2.5 × 10^−2^ Torr. The targets used were ZnO with a purity of 99.99 %, Mn, and Co, both with a purity of 99.9 % (762 mm diameter and 3 mm thickness), positioned 70 mm away from the substrate. The target power for Mn and Co (P_Mn_ and P_Co_, respectively) ranged from 25 W to 50 W, and the substrate temperature varied between 293 K and 423 K, while the target power for ZnO (P_ZnO_) was set to 100 W. All samples were subjected to in situ annealing at 473 K for 2 h.

Structural characterization of the samples was performed via X Ray Diffraction (XRD) measurements using an X-ray diffractometer X'Pert Pro-polycrystal PANalytical, equipped with a source of Cu-Ka 1.540598 Å, a potential difference of 40 kV, current of 40 mA, and X'Celerator detector, in Bragg-Brentano configuration.

Morphological properties were studied through Scanning Electron Microscopy (SEM), and Atomic Force Microscopy (AFM) measurements. The scanning electron microscope was Vega3 TESCAN operating at an acceleration voltage of 10 kV under high vacuum (∼10^−6^ mbar). This microscope has a maximum resolution of 30 nm applying 30 kV in high vacuum and has the EDXS detector system, XFlash® 7, which allows obtaining spectra for compositional analysis. AFM measurements were performed with Asylum Research MFP-3D-BiIO equipment in tapping mode. Measurements I-V were obtained using a picoammeter Keithley 2460 SourceMeter SMU Instrument with Remote Lan 1588 Interlock, at room temperature and atmospheric pressure. Additionally, the magnetic properties of the samples were determined using the VersaLab magnetic property measurements system, based on the vibrating sample magnetometer (VSM) technology.

## 3 Results and discussion

**Fig. 1:**
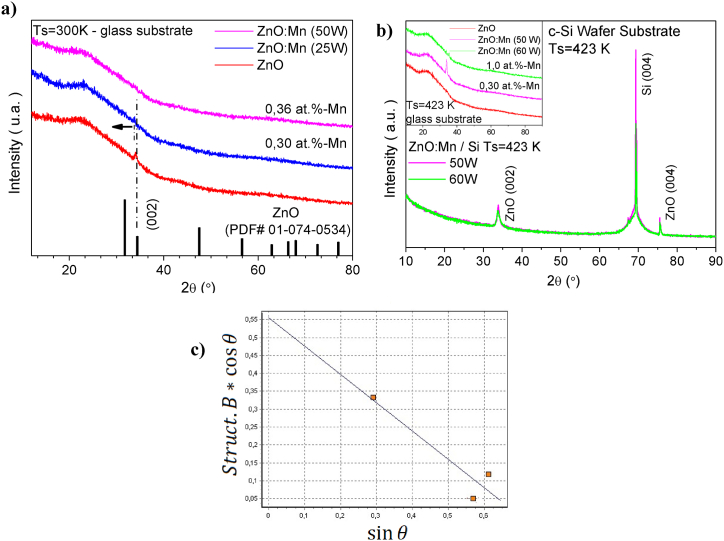
X-ray diffraction patterns in Bragg- Brentano configuration. a) ZnO and ZnO:Mn on glass substrate at Ts = 300 K varying Mn concentration. b) ZnO:Mn on oriented Si wafer at Ts = 423 K varying Mn concentration. The inset shows samples deposited on glass substrate at Ts = 300 K. c) Williamson-Hall plot of ZnO:Mn on oriented Si wafer at Ts = 423 K with 50 W.

In Fig. 1-(a,b), the XRD patterns of the ZnO and ZnO:Mn samples are presented, showcasing variations in the power of the Mn target and the deposition temperature. In Fig. 1-b inset, XRD patterns for the same samples are presented, deposited on a Si wafer substrate while maintaining a temperature of 423 K. The XRD patterns of the deposited thin films presented an amorphous and polycrystalline nature; this characteristic is predominant when a glass substrate is used. This condition is expected due to the amorphous nature of the glass substrate and its influence on layers deposited with thicknesses around of 60 nm and 215 nm.

Upon analyzing the crystalline structure of a pure ZnO film (Fig. 1-a red line), deposited without Mn atoms as a dopant, and comparing its pattern with the one from PDF file 01-074-0534, a hexagonal Wurtzite type structure was identified (P6_3_ mc group), exhibiting only the (002) peak for ZnO at 2θ = 34.24° position, without the formation of secondary phases. A slight increase in crystallinity was observed with the inclusion of Mn atoms in the ZnO matrix, specifically, from ZnO to ZnO:Mn (25 W). A higher increase in Mn target power resulted in the deterioration of crystal formation. The substrate temperature allowed an improvement in the organization of the structure and with a low Mn target power it was possible to observe a small organization in the growth of the material.

Furthermore, comparing the ZnO film and the one doped with Mn, the (002) peak of the ZnO:Mn film (25 W, magenta color) shifted towards a smaller angle (2θ = 33.87°) as indicated by the dotted line in the direction of the arrow in Fig. 1-a. This shift is attributed to the substitution of Mn^2+^, whose ionic radius is greater than the radius of the Zn^2+^ ion (Mn^2+^ = 0.80 Å; Zn^2+^ = 0.74 Å) [[25], [26], [27]], increasing the volume of the ZnO unit cell. This observation confirms the incorporation of Mn^2+^ ions into the ZnO semiconductor matrix while maintaining its crystalline structure. The consistency and reliability of this observation are further supported by the refinement of the XRD data, as indicated by the low *Rwp* value of 1.54. This low *Rwp* reflects a very good fit between the experimental and theoretical patterns, reinforcing the validity of the structural model and confirming that the doping process does not introduce other phases, but rather leads to an orderly incorporation of Mn^2^⁺ into the ZnO lattice.

Considering the identified peaks of the ZnO phase and the Si wafer (see Fig. 1b), a Williamson-Hall plot was utilized to determine the crystallite size and stress in the ZnO:Mn sample (see Fig. 1c). For the sample prepared with 50 W power applied to the Mn target, a compressive stress value of −0.345 % was determined. This negative stress suggests that the interatomic distances in the ZnO crystal lattice were reduced compared to their natural state, likely due to compression induced by the mismatch in thermal expansion coefficients and the lattice parameters between ZnO and the Si substrate. Such stress can also arise from intrinsic defects like oxygen vacancies or extrinsic factors related to fabrication conditions, as supported by prior studies [[28], [29], [30]].

For the ZnO:Mn sample fabricated with 60 W target power, a significantly higher compressive stress of −2.03 % was observed. This increase in stress may be attributed to the elevated Mn concentration, which likely disturbs the ZnO lattice further [30]. Higher doping levels can exacerbate lattice strain by introducing more defects or interstitial atoms, leading to increased structural deformation and compressive forces. This behavior aligns with observations of stress variations due to substrate type and temperature, which directly influence the growth dynamics and defect formation in ZnO thin films [28,29].

The lower concentration of Mn (50W) in the sample appears to allow the growth of larger crystallites without inducing significant stresses in the crystal lattice, as it exhibits a crystallite size of 158.90 Å. This may be due to a lower number of defects or dislocations introduced by doping, allowing for more uniform crystallite growth. In contrast, the sample with a higher concentration of Mn (60W) is causing greater compression in the crystal structure, which may induce a reduction in crystallite size (31.04 Å). This could be the result of the introduction of defects, internal stresses, or changes in the crystal lattice structure due to Mn doping.

**Fig. 2:**
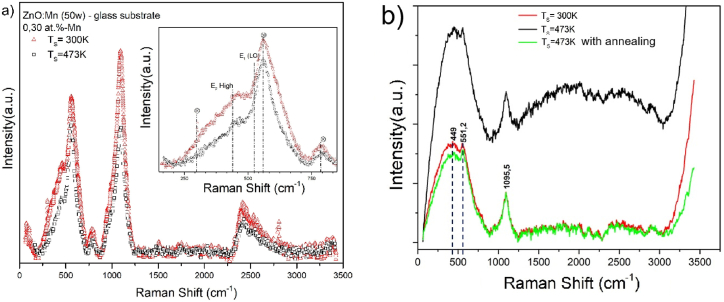
Raman spectra of a) ZnO:Mn thin films deposited at 300K and 473 K on glass substrate varying the Mn concentration. The inset shows high and optical longitude vibrational modes of Raman Shift. b) ZnO:Co thin films at 300K, 473 K, and 473 K with annealing process on glass substrate.

A comparable effect on the crystallinity of the material was observed with the incorporation of Co atoms in the ZnO nanostructure for low target powers. The absence of secondary phases formed by the addition of Mn/Co in the ZnO matrix and its correlation with the XRD patterns were corroborated through Raman spectroscopy measurements (see Fig. 2).

The vibrational modes of the thin films during the measurement process confirm once again that these films exhibit a degree of in-plane orientation (002). Bands associated with vibrational modes attributed to the E2(high) and E1(LO) modes were identified at shifts of 435 cm^−1^ and 530 cm^−1^, respectively. The peak associated with the band at approximately 435 cm^−1^ is considered the characteristic band of the ZnO phase in the samples, indicating that they maintain the ZnO wurtzite structure [27,31]. The additional peaks marked in the figure at 300, 530, and 787 cm^−1^ are attributed to modes of the predominant silicon in the borosilicate substrate.

Consequently, in Fig. 2b for the ZnO:Co thin films, it is possible to observe the peak associated with the band located around 449 cm^−1^ is related to the high-frequency E2 mode, corresponding to the wurtzite phase of ZnO and the tetrahedral configuration of the oxygen atoms. The peak at 551 cm^−1^ is associated with possible defects due to oxygen vacancies, zinc vacancies, or free carriers [27,31]. Additionally, the peak observed at 551 cm^−1^ may be attributed to the E1 (LO) mode, which is associated with structural defects in the synthesized ZnO samples. These defects can be to include oxygen vacancies and zinc interstitials, which play a critical role in influencing the optical and electronic properties of the material. The presence of this peak provides evidence of intrinsic defect states that can affect carrier concentration and recombination dynamics within the ZnO lattice [32].

**Fig. 3:**
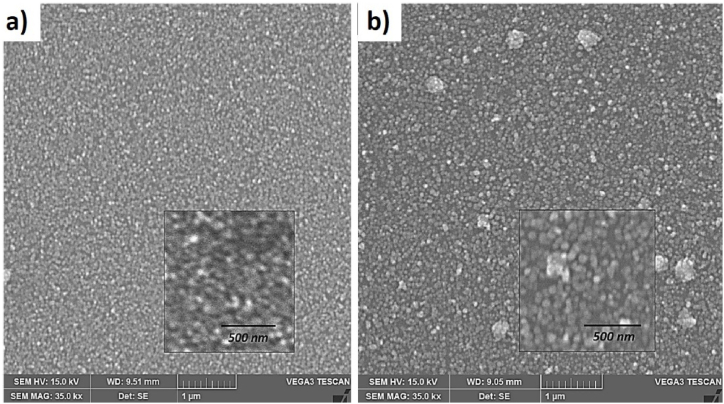
SEM measurements of ZnO:Mn thin films deposited at 300K and 473 K on glass substrate varying the Mn concentration. The inset shows an SEM micrograph with 500 nm of resolution.

The increase in the power of the Mn target enhances the size and ordering of the grains on the surface, as well as a more defined grain boundary. From the SEM micrographs in Fig. 3, it was determined that the grain size is approximately 28.77 ± 7 0.98 nm and 60.76 ± 13.09 nm for the samples with Mn at 50 W and 60 W, respectively. The increase in Mn power leads to the formation of clusters on the surface of the thin films, with an elemental concentration of Mn obtained at 0.99 wt% (of the total detected elements, including the substrate) when the power applied to the target was 60 W (see Fig. 3-a (300K) and Fig. 3-b (473K)). In these clusters, the localized increase in material at certain sites on the surface is evident, which can affect the semiconductor properties of ZnO and additionally cause magnetic phases that oppose the formation of a DMS.

Additionally, EDXS measurements were performed to quantify the atomic weight percentage of Mn present in the ZnO:Mn films, where the concentration was varied based on the target power during deposition. Table 1 presents the results obtained from these spectra.

**Table 1:** wt% of elements identified through EDXS measurements of ZnO:Mn thin films.

Element	Series	ZnO:Mn (50W)	ZnO:Mn (60W)
		C. Norm. (wt%)	C. Norm. (wt%)
O	K-series	91.42	84.25
Zn	K-series	6.74	10.05
Mn	K-series	1.85	5.69

**Fig. 4:**
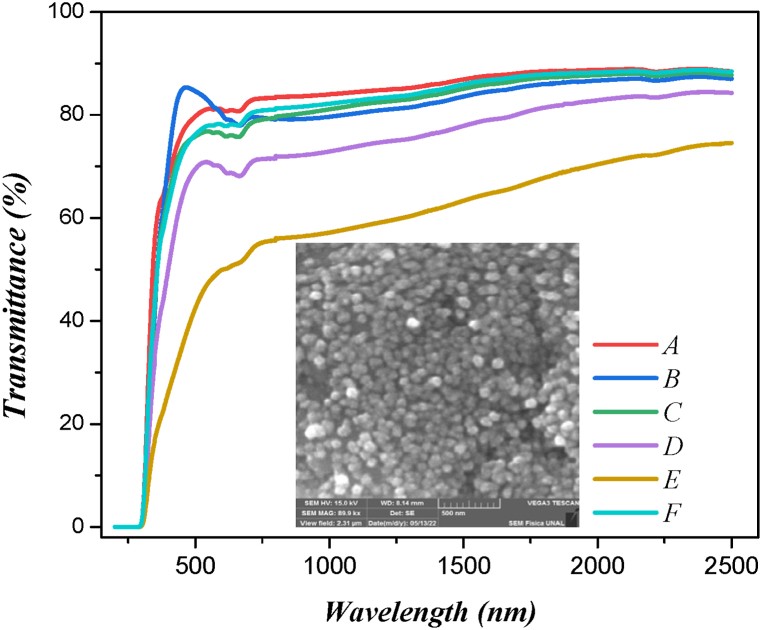
Optical transmittance spectra of ZnO:Co deposited varying the synthesis parameters: Target power, substrate temperature, deposit time, and annealing time (reported Table 1). The inset shows an SEM image of ZnO:Co deposited at 473 K and an annealing temperature in situ of 473 K (sample F).

The transmittance spectra were obtained for variations in Co target magnetron power, deposition time, substrate temperature, and annealing temperature (Table 2). From Fig. 4, it is possible to observe that samples obtained with a substrate temperature of 473 K and in-situ annealing exhibit higher transmittance compared to samples deposited at room temperature. The local maxima and minima of the spectra are attributed to interference conditions from the homogeneity of all the samples [33]. The samples' values of α (absorption coefficient) and energy gap were determined using the Beer-Lambert Law [34].

**Table 2:** Synthesis parameters of ZnO:Co samples.

Sample	ZnO Power (W)	Co Power (W)	Time (min)	Ts (K) Substrate Temperature	Ta (K) Annealing Temperature at 2h	$E_{g}\pm 0,02\;eV$
A	100	25	10	300	/	3,58
B	100	25	15	300	/	3,37
C	100	25	10	473	/	3,44
D	100	50	10	300	/	3,13
E	100	50	15	300	/	3,00
F	100	25	10	473	473	3,42

The estimated values are presented in Table 2, showing that the synthesis parameters significantly influenced the optical bandgap (Eg) of the samples. This value is consistent with reported bandgap values for ZnO in its pure state, typically ranging from 3.2 to 3.3 eV. The results also reveal that the variation in Co target power affects the bandgap of the films. Specifically, at a Co target power of 50 W, the bandgap values are lower than those obtained with a power of 25 W. This observation suggests that increasing the concentration of the dopant material can elevate the carrier concentration (electrons or holes) in the doped ZnO matrix. However, in this case, the reduction in the bandgap with increasing Co concentration could indicate the introduction of localized states within the band structure or enhanced defect levels, which may act as recombination centers, effectively narrowing the observed optical bandgap.

**Fig. 5:**
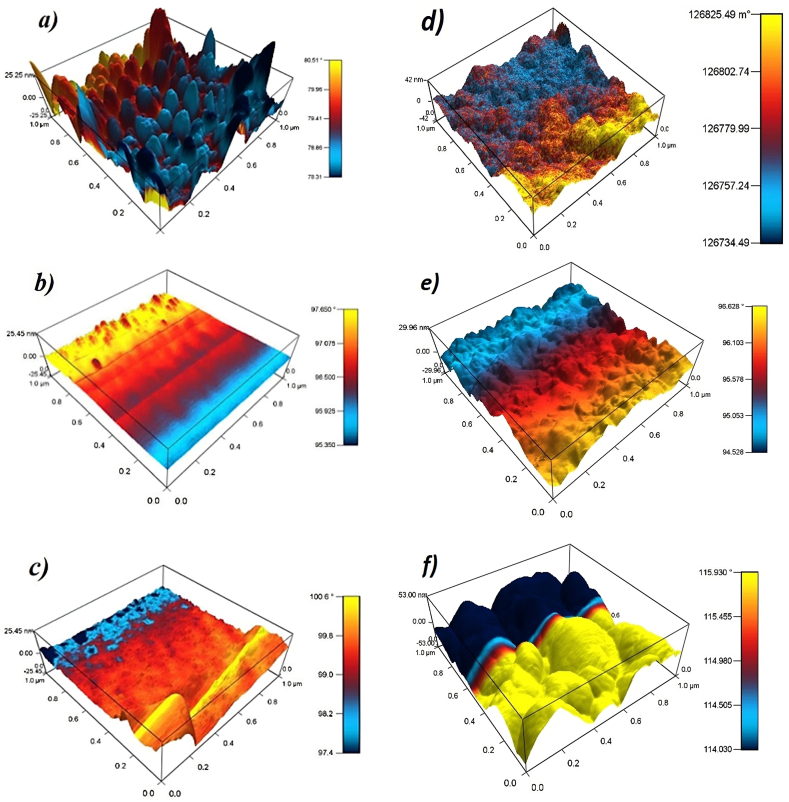
MFM micrographs of ZnO:Mn with a)Ts = 300K, b) Ts = 423K, and c) Ts = 473K. ZnO:Co with d)Ts = 300 K, e) Ts = 473 K, and f) Ts = 473 K and Ta = 473 K. Power of Mn/Co was 25 W, for both cases.

Magnetic force microscopy measurements (MFM) were conducted to identify the magnetic orientation associated with possible magnetic domains. Fig. 5 presents the MFM images using a color scale ranging from warm to cool colors (from yellow to blue).

In Fig. 5-a, a correlation between surface roughness and the attraction or repulsion of the tip on the surface is observed in the sample manufactured at Ts = 300 K with P(Mn) = 25 W; in this case, it is evident that Mn atoms on the surface induce repulsion in the higher areas or regions further away from it. As the temperature increases (see Fig. 5-b (Ts = 423 K) and Fig. 5-c (Ts = 473 K)), Mn concentration configures within the semiconductor matrix, as evidenced in the structural analysis. However, in the MFM measurements, a significant contribution from the glass substrate is apparent.

Likewise, in the case of the ZnO matrix with a concentration of Co atoms, an arrangement of these atoms on the surface and the potential formation of magnetic domains characterized by the presence of Co atoms was observed (see Fig. 5d and e). Fig. 5-f provides evidence of nucleation processes and domain reorganization following post-annealing at 473K for 2 h.

**Fig. 6:**
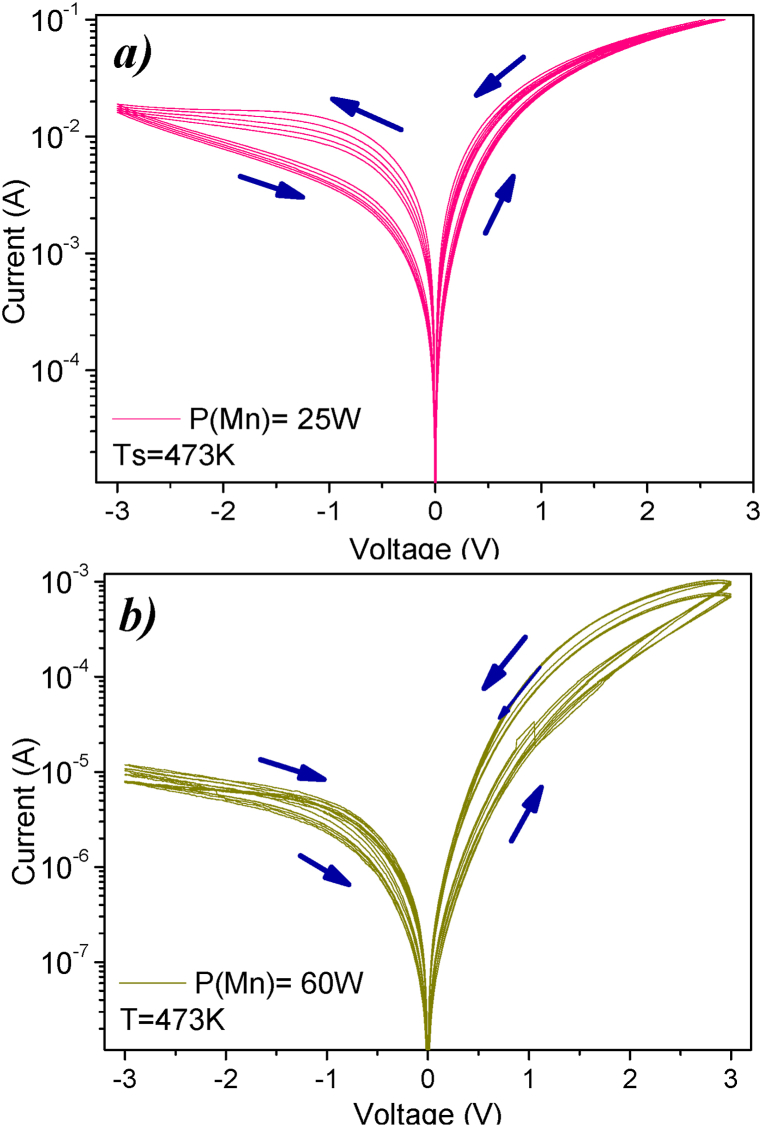
I-V curves of ZnO:Mn samples with Ts = 473 K, varying the concentration of Mn. a) P(Mn) = 25 W, b) P(Mn) = 60 W. The arrows indicate the direction of the current vs. voltage relationship.

Fig. 6 shows the phenomenon of bipolar RS, governed by an interfacial conduction defined by the influence of the applied potential or majority charge carriers. A reduction in the concentration of Mn (power target 25W) promoted RS. In this case, resistive changes are observed from 91.33 Ω to 25.38 Ω during the SET process, with a VSET = 2.538 V for an I_CC_ = 100 mA. During the RESET process, there is a gradual resistive change resulting in a HRS ∼220 Ω at a voltage of −0.08 V. Conversely, for a higher concentration of Mn (Fig. 6-a) the HRS value is higher by 4 orders of magnitude (∼10^6^ Ω) and in LRS it is ∼2.7 × 10^3^ Ω, with V_SET_ = 2.7 V, correspondig to I_optimal_ = 1 mA.

The voltage supplied to the sample also plays a crucial role in the resistive behavior of the films [35]. With variations in voltage, the ZnO:Mn sample (P(Mn) = 60W) exhibited RS with a unipolar trend for voltages of ±1 V and bipolar behavior for ± 2 V and ±3 V, along with a decrease in LRS (Fig. 6-b).

**Fig. 7:**
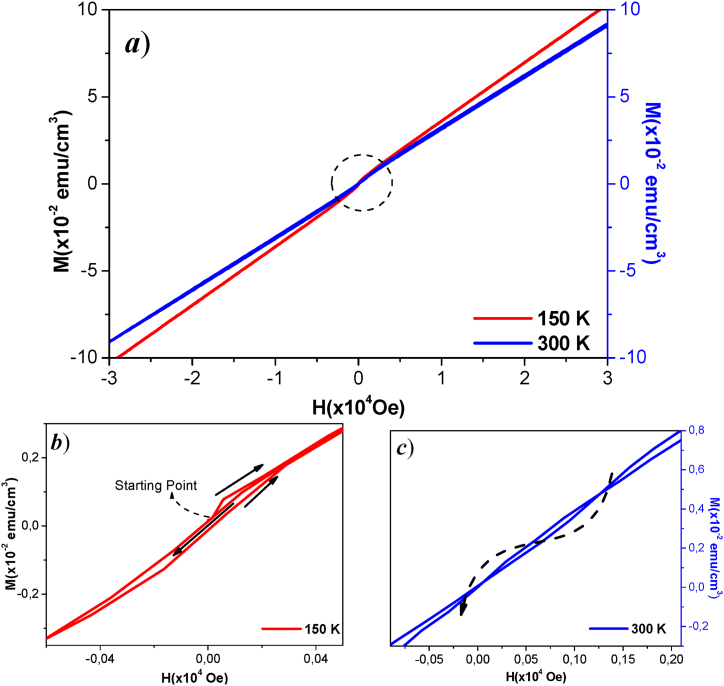
M vs H of ZnO:Mn, P(Mn) = 25 W, Ts = 423 K when a) T_M_ = 150 K (red) and T_M_ = 300 K (blue). Expanded view of the hysteresis: b) M vs H at T_M_ = 150 K, c) M vs H at T_M_ = 300 K (dashed arrow as visual support of the direction of the curve).

Fig. 7a shows M vs H for a ZnO sample deposited at Ts = 423 K, with 25 W power of Mn (0.02 wt%), displaying a hysteresis trend. In Fig. 7-b, a slight opening in the hysteresis is visible, with coercive field $H_{C}=-18,76\;Oe$ and remanent magnetization of $M_{R}=1,35x10^{-4}\frac{emu}{{cm}^{3}}$ (150 K) and $M_{R}=-1.26x10^{-4}\frac{emu}{{cm}^{3}}$ (300 K) (see Fig. 7-c).

The incorporation of Mn atoms in the ZnO semiconductor matrix contributes to this behavior, associated with unpaired electrons in the 3d layer [36]. As the magnetization temperature (T_M_, = 300K) increases, the sample exhibits behavior associated with moments of order and disorder of the spin of itinerant electrons, fluctuating between ferromagnetic and paramagnetic states. This behavior, causes a late response in the magnetic ordering with the variation of the magnetic field H.

As the magnetization temperature (TM = 300K) increases, the sample with low Mn concentration within ZnO exhibits behavior indicative of the interplay between ordered and disordered moments of itinerant electron spins. At this temperature, the material fluctuates between ferromagnetic and paramagnetic states, reflecting a complex magnetic response.

In low Mn doping, the interaction between the Mn ions and the ZnO matrix influences the overall magnetic properties. At low concentrations, the Mn ions introduce localized magnetic moments, but their effect is less pronounced due to limited interaction with the surrounding ZnO lattice [27]. This results in a situation where the thermal energy at elevated temperatures disrupts the alignment of these localized moments, causing a fluctuation between ferromagnetic ordering and paramagnetic behavior [[37], [38],^39^].

Therefore, ferromagnetic state in ZnO:Mn originates mainly due to the interaction between Mn atoms, oxygen vacancies (*V*_*O*_), and oxygen atoms. The presence of oxygen vacancies stabilizes the ferromagnetic configuration, resulting in a higher Curie temperature [36]. The electronic structure shows that the ferromagnetic phase is the most stable configuration when oxygen vacancies are present in the structure. The presence of oxygen vacancies also leads to a decrease in the average magnetic moment per unit cell, which is observed in the spatial distribution of the spin density. These results suggest that oxygen vacancies in ZnO:Mn are key to achieving stable ferromagnetism in this diluted magnetic semiconductor, with potential applications in spintronic devices [38].

Adittionally, the presence of Mn introduces additional magnetic interactions within the ZnO matrix. However, at low doping levels, these interactions are insufficient to establish a strong, stable ferromagnetic order. Instead, the sample demonstrates a more dynamic magnetic response, with spins exhibiting a mix of ferromagnetic alignment and random paramagnetic orientations. This behavior results in a delayed or non-linear magnetic ordering response when subjected to external magnetic field variations (H).

Specifically, as the external field is applied or varied, the magnetic response of the sample becomes more complex and less predictable due to the competing influences of thermal agitation and the weak magnetic interactions introduced by the low Mn concentration. This intricate balance between thermal energy and the limited magnetic interaction highlights the challenges in achieving robust magnetic ordering in Mn-doped ZnO systems with low dopant levels [40].

## 4 Conclusions

Through FESEM and AFM microscopy, the topography of the samples was identified, ZnO:Mn/Co, was identified, primarily, composed of small granules depending on the synthesis parameters. In the case of ZnO:Mn grains of the order of ∼12.16 ± 2.69 nm to 60.75 ± 13.09 nm were observed. A reduction in the size of the grains and the boundary between them was observed with increasing temperature, with a minor influence on the concentration of Co/Mn atoms in the ZnO matrix. Significant improvement in the domain regions present in the ZnO material was attributed to the atoms incorporated into the host matrix. The electrical study identified samples exhibiting unipolar and bipolar resistive switching (RS) behavior, along with non-filamentary or interfacial conductivity, interpreted through the Schottky barrier model. The attraction or repulsion of oxygen vacancies near the contact widens or narrows the width of the barrier, allowing charge flow and RS under a bias voltage. Vibrating sample magnetometry (VSM) revealed hysteresis associated with ferromagnetism for samples deposited with a substrate temperature Ts = 423 K, Mn power 25 W, and Co power 50 W. The obtained information confirms the feasibility of fabricating dilute magnetic semiconductors (DMS) from ZnO:Mn/Co with potential applications in spintronic devices.

## CRediT authorship contribution statement

**Ángela P. Lanchero:** Methodology, Investigation, Formal analysis, Data curation. **Lina F. Prieto:** Methodology, Investigation, Formal analysis, Data curation, Conceptualization. **Heiddy P. Quiroz:** Supervision, Resources, Methodology, Investigation, Formal analysis, Data curation. **Jorge A. Calderón:** Supervision, Resources, Methodology, Investigation, Formal analysis, Data curation, Conceptualization. **A. Dussan:** Writing – review & editing, Writing – original draft, Validation, Supervision, Project administration, Methodology, Funding acquisition, Formal analysis, Data curation. **F. Mesa:** Writing – review & editing, Writing – original draft, Validation, Supervision, Investigation, Funding acquisition, Formal analysis, Conceptualization.

## Data availability statement

Data included in the article/supplementary material is referenced in the article.

## Declaration of competing interest

The authors declare that they have no known competing financial interests or personal relationships that could have appeared to influence the work reported in this paper.

## References

[1] Achouri F., Corbel S., Balan L., Mozet K., Girot E., Medjahdi G., Said M.B., Ghrabi A., Schneider R. (2016). Porous Mn-doped ZnO nanoparticles for enhanced solar and visible light photocatalysis. Mater. Des..

[2] Tian Y.F., Bakaul S.R., Wu T. (2012). Oxide nanowires for spintronics: materials and devices. Nanoscale.

[3] Jabbar I. (2022). Diluted magnetic semiconductor properties in TM doped ZnO nanoparticles. RSC Adv..

[4] Khan R. (2022). Room temperature dilute magnetic semiconductor response in (Gd, Co) co-doped ZnO for efficient spintronics applications. RSC Adv..

[5] Khan R., Fashu S., ur Rehman Z. (2017). Structural, dielectric and magnetic properties of (Al, Ni) co-doped ZnO nanoparticles. J. Mater. Sci. Mater. Electron..

[6] Phan T.L., Zhang P., Yang D.S., Nghia N.X., Yu S.C. (2011). Local structure and paramagnetic properties of Zn_1-x_Mn_x_O. J. Appl. Phys..

[7] Dietl T., Ohno H., Matsukura F., Cibert J., Ferrand D. (2000). Zener model description of ferromagnetism in zinc-blende magnetic semiconductors. Science.

[8] Calderón Jorge A., Terán Cristian L., Quiroz Heiddy P., Dussan A., Manso-Silván M. (2023). Ion migration in GaSb/Mn multilayers for memories applications: study of Mn diffusion into the GaSb layers. J. Alloys Compd..

[9] Murtaza Adil, Zuo Wen-liang, Song Xianghao, Ghani Awais, Saeed Azhar, Yaseen Muhammad, Tian Fanghua, Yang Sen (2022). Robust ferromagnetism in rare-earth and transition metal co-doped ZnO nanoparticles for spintronics applications. Mater. Lett..

[10] Pearton S.J., Norton D.P., Heo Y.W. (2006). ZnO spintronics and nanowire devices. J. Electron. Mater..

[11] Ullah A. (2023). Effect of yttrium on the structural, dielectric, and magnetic properties of Co-doped ZnO magnetic nanorods for potential spintronic applications. J. Mater. Sci. Mater. Electron..

[12] Raha Sauvik, Ahmaruzzaman Md (2022). ZnO nanostructured materials and their potential applications: progress, challenges and perspectives. Nanoscale Adv..

[13] Shatnawi M., Alsmadi A.M., Bsoul I., Salameh B., Mathai M., Alnawashi G., Alzoubi Gassem M., Al-Dweri F., Bawaaneh M.S. (2016). Nfluence of Mn doping on the magnetic and optical properties of ZnO nanocrystalline particles. Results Phys..

[14] Ahmed S.A. (2017). Structural, optical, and magnetic properties of Mn-doped ZnO samples. Results Phys..

[15] Singh Karanpal, Nancy, Kaur Harpreet, Sharma Pushpender Kumar, Singh Gurjinder, Singh Jagpreet (2023). ZnO and cobalt decorated ZnO NPs: synthesis, photocatalysis and antimicrobial applications. Chemosphere.

[16] Peña-Garcia R., Guerra Y., Castro-Lopes S., Camejo Y.M., Soares João M., Franco A., Padrón-Hernández E., Cabrera-Baez M. (2021). Morphological, magnetic and EPR studies of ZnO nanostructures doped and co-doped with Ni and Sr. Ceram. Int..

[17] Lins A., Jerônimo A.G., Barbosa R., Neves L., Trigueiro P., Almeida L.C., Osajima J.A., Pereira F.A., Peña-Garcia R.R. (2023). Facile synthesis of Ni-doped ZnO nanoparticles using cashew gum: investigation of the structural, optical, and photocatalytic properties. Molecules.

[18] Cabrera-Baez M., Padrón-Hernández E., Soares João M., Santos F.E.P., Guerra Y., Peña-Garcia R. (2021). Effect of yttrium substitution in Fe-doped ZnO nanoparticles: an EPR study. J. Magn. Magn Mater..

[19] Silva M.C.R., Castro-Lopes S., Jerônimo A.G., Barbosa R., Lins A., Trigueiro P., Viana B.C., Araujo F.P., Osajima J.A., Peña-Garcia R.R. (2024). Green synthesis of Er-doped ZnO nanoparticles: an investigation on the methylene blue, eosin, and ibuprofen removal by photodegradation. Molecules.

[20] Gu Zheng-bin, Lu Ming-hui, Wang Jing, Du Chao-ling, Yuan Chang-sheng, Wu Di, Zhang Shan-tao, Zhu Yong-yuan, Zhu Shi-ning, Chen Yan-feng (2006). Optical properties of (Mn, Co) co-doped ZnO films prepared by dual-radio frequency magnetron sputtering. Thin Solid Films.

[21] Morkoç Hadis, Özgür Ümit (2009).

[22] Sharma D., Jha R. (2017). Transition metal (Co, Mn) co-doped ZnO nanoparticles: effect on structural and optical properties. J. Alloys Compd..

[23] Nirmala M., Smitha P., Anukaliani A. (2011). Optical and electrical properties of undoped and (Mn, Co) co-doped ZnO nanoparticles synthesized by DC thermal plasma method. Superlattice. Microst..

[24] Sharma V.K., Najim M., Srivastava A.K., Varma y G.D. (2012). Structural and magnetic studies on transition metal (Mn, Co) doped ZnO nanoparticles. J. Magn. Magn Mater..

[25] Lekoui F. (2021). Investigation of the effects of thermal annealing on the structural, morphological and optical properties of nanostructured Mn doped ZnO thin films. Opt. Mater..

[26] Ruan H.B., Fang L., Li D.C. (2011). Effect of dopant concentration on the structural, electrical and optical properties of Mn-doped ZnO films. Thin Solid Films.

[27] Yang S., Zhang Y. (2013). Structural, optical and magnetic properties of Mn-doped ZnO thin films prepared by sol-gel method. J. Magn. Mater..

[28] Puthiyottil Hajara, Rose Thankamani Priya, Joseph Saji Kachirayil (2023). Exploring the effects of substrate and substrate temperature on the properties of radio frequency magnetron sputtered ZnO thin films. Mater. Today Commun..

[29] Lekoui Fouaz, Amrani Rachid, Hassani Salim, Garoudja Elyes, Filali Walid, Ouchabane Mohammed, Hendaoui Nordine, Oussalah Slimane (2024). On the substrate heating effects on structural, mechanical and linear/non-linear optical properties of Ag–Mn co-doped ZnO thin films. Opt. Mater..

[30] Joshi P., Singh J., Jain V.K., Akhtar J.A., Ledwani L., Sangwai J. (2020). Nanotechnology for Energy and Environmental Engineering.

[31] Zhuo R.F., Fen H.T., Liang Q., Liu J.Z., Chen J.T., Yan D., Feng J.J., Li H.J., Cheng S., Geng B.S., Xu X.Y., Wang J., Wu Z.G., Yan P.X., Yue G.H. (2008). Morphology-controlled synthesis, growth mechanism, optical and microwave absorption properties of ZnO nanocombs. J. Phys. D Appl. Phys..

[32] Soares A.S., Araujo Francisca P., Osajima Josy A., Guerra Y., Viana Bartolomeu C., Peña-Garcia R. (2024). Nanotubes/nanorods-like structures of La-doped ZnO for degradation of methylene blue and ciprofloxacin. J. Photochem. Photobiol. Chem..

[33] Terán C.L., Calderón Jorge A., Quiroz Heiddy P., Dussan A. (2021). Optical properties and bipolar resistive switching of ZnO thin films deposited via DC magnetron sputtering. Chin. J. Phys..

[34] Delgado R. (2022). Misuse of Beer–Lambert Law and other calibration curves. R. Soc. Open Sci..

[35] Sawa A. (2008). Resistive switching in transition metal oxides. Mater. Today.

[36] Sharma M., Bera K., Mishra R. (2021). Structural, magnetic, and optical properties of Mn2+ doping in ZnO thin films. Surfaces.

[37] Galindez E.F., Mendoza-Estrada V., González-García A., López-Pérez W., González-Hernández R., Dussan A. (2022). Influence of oxygen vacancies on the ferromagnetism in Co-doped ZnO: an ab-initio study. Solid State Commun..

[38] Deepika Raju Kumar, Kumar Ritesh, Prasad Yadav Kamdeo, Vaibhav Pratyush, Sharma Seema, Singh Rakesh Kumar, Kumar Santosh (2020). Defect induced room-temperature ferromagnetism and enhanced photocatalytic activity in Ni-doped ZnO synthesized by electrodeposition. Chin. Phys. B.

[39] Motaung D.E., Mhlongo G.H., Nkosi S.S., Malgas G.F., Mwakikunga B.W., Coetsee E., Swart H.C., Abdallah H.M.I., Moyo T., Ray S.S. (2014). Shape-selective dependence of room temperature ferromagnetism induced by hierarchical ZnO nanostructures. ACS Appl. Mater. Interfaces.

[40] Taalab Z., Amer M.I., Moustafa S.H., Hashem H.M., Emam- Ismail M., Shaaban E.R., Hammam M., El-Hagary M. (2024). Exploring enhanced optoelectronic and spintronic characteristics of Fe-doped ZnO nanoparticles synthesized by co-precipitation approach. Mater. Sci. Semicond. Process..

